# Mianserin suppresses R-spondin 2-induced activation of Wnt/β-catenin signaling in chondrocytes and prevents cartilage degradation in a rat model of osteoarthritis

**DOI:** 10.1038/s41598-019-39393-x

**Published:** 2019-02-26

**Authors:** Toshiaki Okura, Bisei Ohkawara, Yasuhiko Takegami, Mikako Ito, Akio Masuda, Taisuke Seki, Naoki Ishiguro, Kinji Ohno

**Affiliations:** 1 Division of Neurogenetics, Center for Neurological Diseases and Cancer, Nagoya University Graduate School of Medicine, Nagoya, Japan; 2 Department of Orthopedic Surgery, Nagoya University Graduate School of Medicine, Nagoya, Japan

## Abstract

Aberrant activation of the Wnt/β-catenin signaling pathway promotes the progression of osteoarthritis (OA). We previously reported that R-spondin 2 (Rspo2), an activator of the Wnt/β-catenin signaling, facilitates differentiation of proliferating chondrocytes into hypertrophic chondrocytes by enhancing Wnt/β-catenin signaling in endochondral ossification. However, the role of Rspo2 in OA remains elusive. Here, we showed that the amounts of Rspo2 protein in synovial fluid were increased in OA patients. We searched for a preapproved drug that suppresses Rspo2-induced Wnt/β-catenin signaling in chondrogenic cells and reduces joint pathology in a rat model of OA. In Rspo2-treated ATDC5 cells, mianserin, a tetracyclic antidepressant, inhibited Wnt/β-catenin signaling, increased proteoglycan production, and upregulated chondrogenic marker genes. Mianserin suppressed Rspo2-induced accumulation of β-catenin and phosphorylation of Lrp6. We identified that mianserin blocked binding of Rspo2 to its receptor Lgr5. We also observed that intraarticular administration of mianserin suppressed β-catenin accumulation and prevented OA progression in a rat model of OA. We conclude that mianserin suppresses abnormally activated Wnt/β-catenin signaling in OA by inhibiting binding of Rspo2 to Lgr5.

## Introduction

Osteoarthritis (OA) is characterized by progressive loss of articular cartilage and concomitant loss of extracellular matrix (ECM), and causes pain and functional disorders in elderly people^1,2^. ECM is comprised of a highly hydrated fibrillar network of collagens embedded in a gel of negatively charged proteoglycans like aggrecan (*Acan*)^3^.

Wnt/β-catenin signaling pathway plays crucial roles in determination of cell fate, and controls tissue homeostasis^4^. Binding of secreted Wnts to their cell-surface receptor comprised of Frizzled and low-density lipoprotein receptor-related proteins 5/6 (Lrp5/6) phosphorylates Lrp5/6 and accumulates β-catenin. Accumulated β-catenin is translocated into the nucleus, and interacts with transcriptional factors, T-cell factor/lymphoid enhancer factor (TCF/LEF), to regulate gene expressions. Excessive activity of Wnt/β-catenin signaling has been implicated in many human diseases including OA^5,6^. Activation of Wnt/β-catenin signaling promotes hypertrophic differentiation of articular chondrocytes which, in turn, induces cartilage degradation and subsequent OA aggravation^7^. Indeed, OA progression is mitigated by inhibiting the Wnt/β-catenin signaling^8–10^.

R-spondins (Rspos) were originally identified as secreted positive-feedback activators of Wnt/β-catenin signaling in *Xenopus*^11^ and often co-expressed with Wnts^12^. Rspos binds to two cell-surface receptors: Lgr4-6 (leucine-rich repeat containing G protein-coupled receptors) and RNF43 (ring finger protein 43)/ZNRF3 (zinc and ring finger 3)^13–16^. RNF43/ZNRF3 are E3 ubiquitin ligases that degrade the Wnt receptors, Frizzled and Lrp5/6. Rspos bridge Lgr4-6 and ZNRF3/RNF43, and induce endocytosis of Lgr4-6 and ZNRF3/RNF43, which suppresses the E3 ubiquitin ligase activity of ZNRF3/RNF43^17,18^. The effects of monoclonal antibodies against Rspo1, Rspo2, and Rspo3 to suppress Rspo-induced signaling are reported in human tumor xenografts^19^.

Rspo2 is an important factor for regulating cell proliferation and differentiation, as well as tissue development. Single nucleotide variations in *RSPO2* are associated with proliferative bone and soft tissue diseases in human^20,21^. We recently reported that Rspo2 activates Wnt/β-catenin signaling and reduces expressions of chondrogenic marker genes of *Sox9* (sex-determining region Y-Box 9; a master gene for chondrocyte differentiation), *Col2a1* (collagen type II α1), and *Acan*, which subsequently facilitates differentiation of proliferating chondrocytes into hypertrophic chondrocytes in the growth cartilage^22^. Hypertrophic differentiation of articular chondrocytes is similarly observed in human OA and animal OA models^23,24^. Excessive Wnt/β-catenin signaling activity facilitates hypertrophic differentiation of articular chondrocytes, which, in turn, induces cartilage-degrading metalloproteinase expression and subsequent OA aggravation^7,25^. However, involvement of Rspo2 in OA remains to be elucidated. Here, we observed that Rspo2 is increased in synovial fluid in OA patients. We next searched for a drug to antagonize the effect of Rspo2 using the drug repositioning strategy, in which a drug currently used to treat a specific disease is applied to another disease^26,27^. We found that mianserin, a tetracyclic antidepressant, suppressed Rspo2-induced activation of Wnt/β-catenin signaling in chondrocytes, which subsequently ameliorated loss of proteoglycans and increased expressions of *Sox9*, *Col2a1*, and *Acan*. Mianserin exerted its anti-Rspo2 effect by directly blocking binding of Rspo2 to Lgr5. In a rat model of OA, intraarticular administration of mianserin reduced accumulation of β-catenin in articular chondrocytes and prevented OA progression.

## Results and Discussion

### Rspo2 in synovial fluid in patients with knee OA increases with increasing OA severities

**Figure 1:**
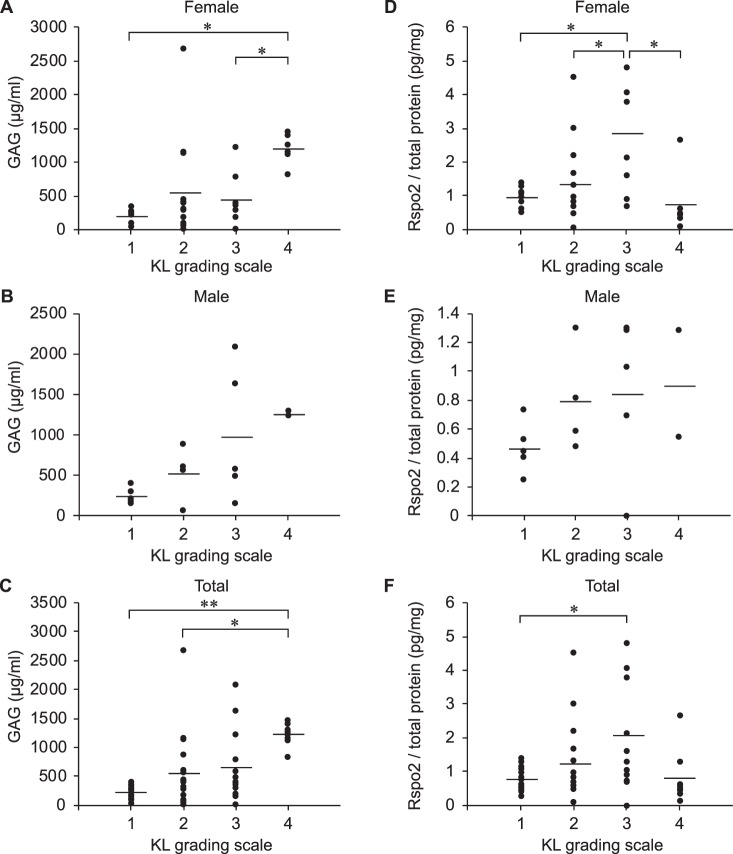
Rspo2 in synovial fluid of knee joints is increased in OA patients with Kellgren-Lawrence (KL) grades 2 and 3. (**A–C)** Concentrations of glycosaminoglycans (GAG) in synovial fluid were measured by a spectrophotometric dye binding assay using 1,2, dimethylmethylene blue (DMMB), and were plotted against the KL grading scale representing OA severity for females (*n* = 36) **(A)**, males (*n* = 16) (**B**), and all patients (*n* = 52) **(C)**. (**D–F)** The amount of Rspo2 was normalized to the amount of total protein in synovial fluid. Rspo2 in females (*n* = 36) **(D)**, males (*n* = 16) (**E**), and all patients (*n* = 52) **(F)** are plotted against the KL grading scale. Bars indicate average values for each KL grade. *P < 0.05 and **P < 0.01 by one-way ANOVA followed by Tukey’s *post-hoc* test. Values of each patient are shown in Supplementary Table S1.

We first confirmed that glycosaminoglycans (GAG), which are degradation products of GAG chains in cartilage proteoglycans^28^, were increased in synovial fluid with increasing OA severities that was evaluated by the Kellgren-Lawrence (KL) radiographic grading system^29^ (Fig. 1A–C). We next observed that intraarticular Rspo2 gradually increased with worsening of OA up to KL grade 3, but was decreased at KL grade 4. This may represent that the number of intraarticular Rspo2-producing and other cells is reduced at KL grade 4. Alternatively, the increase of Rspo2 is associated with only initiation and progression of OA (Fig. 1D–F, Supplementary Table S1). The amounts of Rspo2 were much higher in female patients than male patients (Fig. 1D,E). In contrast to Rspo2, the amounts of Wnt antagonists including dickkopf (DKK)-1, DKK-2, and sclerostin in synovial fluid are inversely correlated with OA severity^30–32^.

### Mianserin inhibits Rspo2-induced activation of Wnt/β-catenin signaling and increases the amounts of Rspo2-reduced ECM in human chondrosarcoma (HCS)-2/8 cells

**Figure 2:**
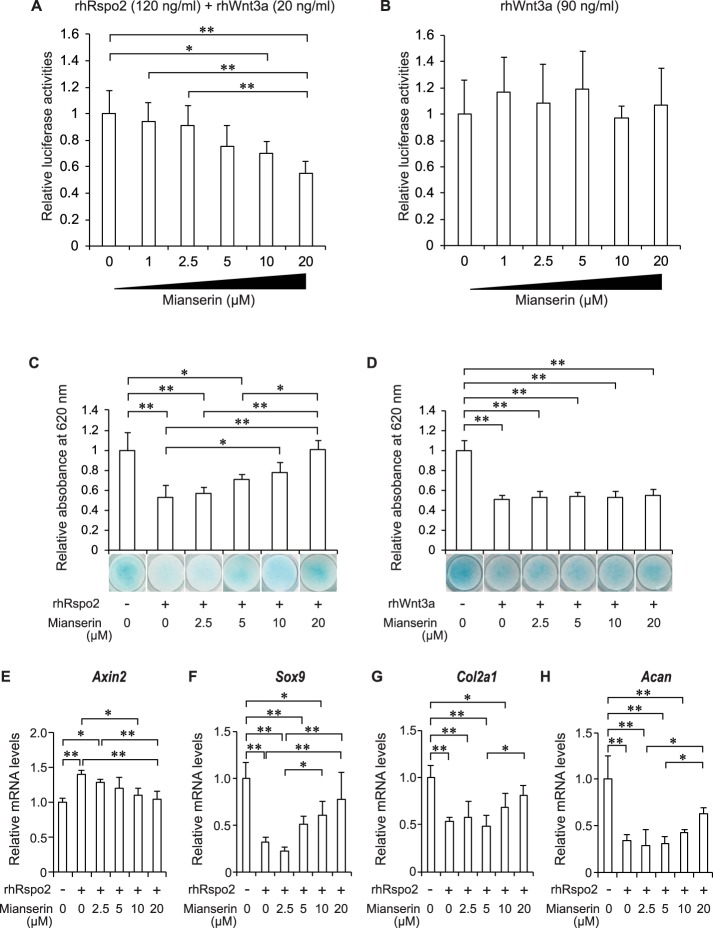
Mianserin suppresses Rspo2-induced activation of Wnt/β-catenin signaling in chondrogenic cell lines. (**A**,**B**) HCS-2/8 human chondrosarcoma cells transfected with the TOPFlash reporter plasmid to quantify Wnt/β-catenin signaling activity were treated with either 120 ng/ml recombinant human Rspo2 (rhRspo2) and 20 ng/ml recombinant human Wnt3a (rhWnt3a) **(A)** or 90 ng/ml rhWnt3a alone (**B**) along with increasing concentrations of mianserin for 24 h. Firefly luciferase activity of the TOPFlash reporter was normalized to the TK promoter-driven Renilla luciferase activity (*n* = 5). (**C**,**D**) ATDC5 cells were cultured with 1% ITS for 2 weeks to induce chondrogenic differentiation and subsequently treated with either 200 ng/ml rhRspo2 **(C**) or 90 ng/ml rhWnt3a **(D**) for 48 h in the presence of the indicated concentrations of mianserin. After staining with Alcian blue, proteoglycans in the cell lysates were quantified by measuring optical densities at 620 nm (*n* = 4). (**E–H**) Chondrogenically differentiated ATDC5 cells were treated with 200 ng/ml rhRspo2 for 48 h in the presence of the indicated concentrations of mianserin. Expression levels of each mRNA were quantified by quantitative RT-PCR, and were normalized for those of *Gapdh* and also for untreated cells (*n* = 4). Mean and SD are indicated. *P < 0.05 and **P < 0.01 by one-way ANOVA followed by Tukey’s *post-hoc* test.

We next attempted to identify a clinically applicable drug that inhibits Rspo2-induced activation of Wnt/β-catenin signaling and OA progression. We quantified Wnt/β-catenin signaling activity using the TOPFlash luciferase reporter assay in the presence of 1,271 FDA-approved drugs in HCS-2/8 cells, and searched for a drug that suppresses Rspo2-activated Wnt/β-catenin signaling. Recombinant human Rspo2 (rhRspo2) alone does not activate Wnt/β-catenin signaling in HCS-2/8 cells, but enhances the signaling in the presence of a low dose of recombinant human Wnt3a (rhWnt3a) (Supplementary Fig. S1A)^17^. We thus performed drug screening with 120 ng/ml rhRspo2 and 20 ng/ml rhWnt3a, and found that a tetracyclic antidepressants (TeCA), mianserin, that is an antagonist or inverse agonist of the histaminergic H_1_ receptor, serotoninergic 5-HT_1–7_ receptors, and α_2_-adrenergic receptor, suppressed the TOPFlash reporter activity in a dose-dependent manner (Fig. 2A). Interestingly, mianserin did not reduce Wnt/β-catenin signaling activated by rhWnt3a alone (Fig. 2B). We observed that 120 ng/ml rhRspo2 and 20 ng/ml rhWnt3a upregulated mRNA expression of Wnt/β-catenin-responsive *AXIN2*^33^ in HCS-2/8 cells, and that mianserin attenuated the upregulation of *AXIN2* (Supplementary Fig. S1B). We also observed similar tendencies in two other Wnt/β-catenin-responsive genes of *CCND1*^34^ and *MYC*^35^ in HCS-2/8 cells (Supplementary Fig. S1B–D). We next tested two other TeCAs: maprotiline, the first TeCA ever to be developed^36^, and mirtazapine, one of analogues for mianserin^37^. In contrast to mianserin, neither maprotiline nor mirtazapine suppressed the TOPFlash reporter activity at 10 μM (1.000 ± 0.272 and 1.018 ± 0.289, mean and SD, *n* = 3, respectively). Taken together, mianserin inhibited the TOPFlash reporter activity via Rspo2, and the effect is unlikely to be associated with its tetracyclic structure.

We evaluated the effects of mianserin on ECM production in mouse chondrogenic ATDC5 cells, which produce high levels of ECM when Wnt/β-catenin signaling is not activated^22^. Quantitative analysis of Alcian blue staining revealed that mianserin ameliorated rhRspo2-induced, but not rhWnt3a-induced, reduction of proteoglycans (Fig. 2C,D). We also confirmed that mianserin mitigated Rpos2-induced upregulation of *Axin2* (Fig. 2E), as well as Rspo2-induced downregulation of *Sox9*, *Col2a1*, and *Acan* (Fig. 2F,G,H). These results indicate that mianserin mitigates Rspo2-induced suppression of ECM production. As far as we know, the effect of mianserin on Rspo2 has not been reported previously. We previously reported that another antidepressant, fluoxetine, ameliorates cartilage degradation in OA by inhibiting Wnt/β-catenin signaling. The putative target of fluoxetine, however, is likely to be a degradation complex including β-catenin or its downstream signaling, and not Rspo2^10^.

### Mianserin reduces Rspo2-induced β-catenin accumulation and Lrp6 phosphorylation, and blocks binding of Rspo2 to Lgr5

**Figure 3:**
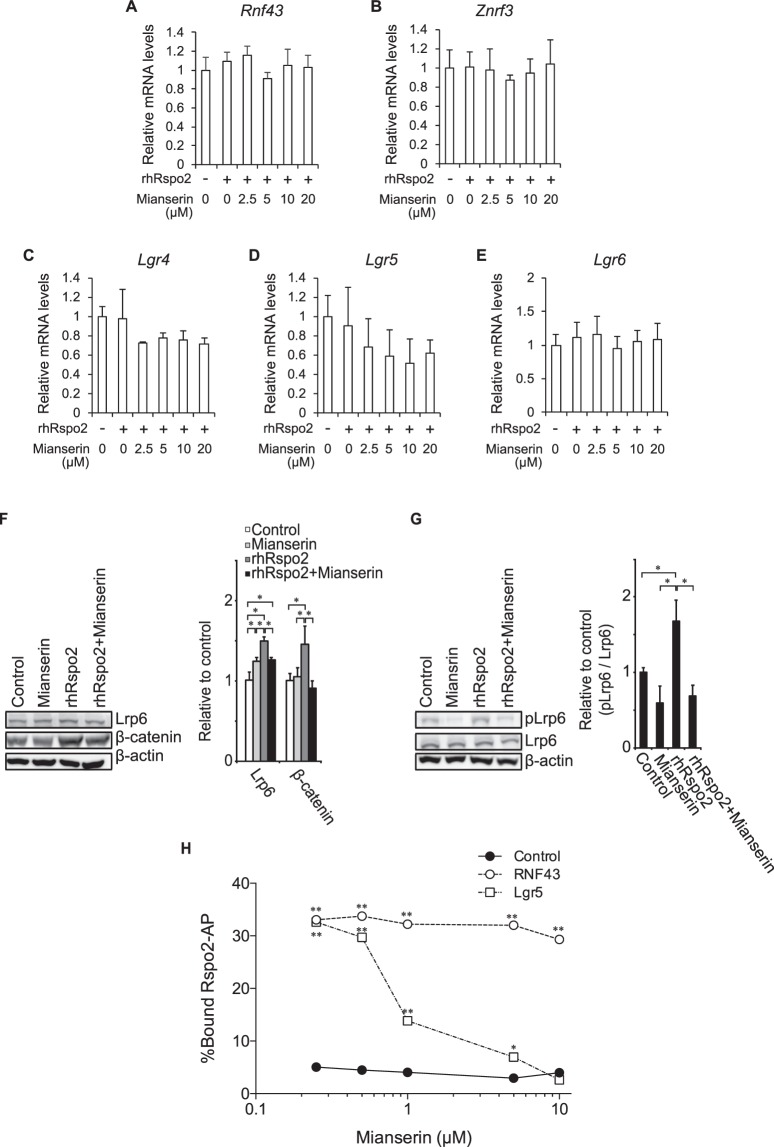
Mianserin reduces Rspo2-induced accumulation of β-catenin and phosphorylation of Lrp6 by suppressing binding of Rspo2 to Lgr5. (**A–E**) ATDC5 cells were cultured with 1% ITS for 2 weeks to induce chondrogenic differentiation and subsequently treated with 200 ng/ml rhRspo2 for 48 h in the presence of the indicated concentrations of mianserin. Expression levels of each mRNA by quantitative RT-PCR were normalized for those of *Gapdh*, and also for untreated cells. Mean and SD are indicated (*n* = 4). No statistical differences were observed by one-way ANOVA followed by Tukey’s *post-hoc* test. (**F**) Differentiated ATDC5 cells were cultured with 10 μM mianserin and 200 ng/ml rhRspo2 for 48 h. Representative Western blots and intensities of Lrp6 and β-catenin normalized for those of β-actin and also for untreated cells are shown. Mean and SD are indicated (*n* = 3). *P < 0.05 by one-way ANOVA followed by Tukey’s post-hoc test. (**G**) Differentiated ATDC5 cells were cultured with 10 μM mianserin and 200 ng/ml rhRspo2 for 1.5 h. Representative Western blots and intensities of phosphorylated Lrp6 normalized for those of total Lrp6 and also for untreated cells are shown. Mean and SD are indicated (*n* = 3). *P < 0.05 by one-way ANOVA followed by Tukey’s post-hoc test. (**H**) Cell surface binding assay of Rspo2-AP in the presence of increasing concentrations of mianserin on HEK293 cells expressing human Lgr5 or RNF43. Bound Rspo2-AP was normalized for the added Rspo2-AP. Mean and SD are indicated (*n* = 3). *P < 0.05 and **P < 0.01 compared to control cells expressing neither Lgr5 nor RNF43 by one-way ANOVA followed by Tukey’s *post-hoc* test.

**Figure 4:**
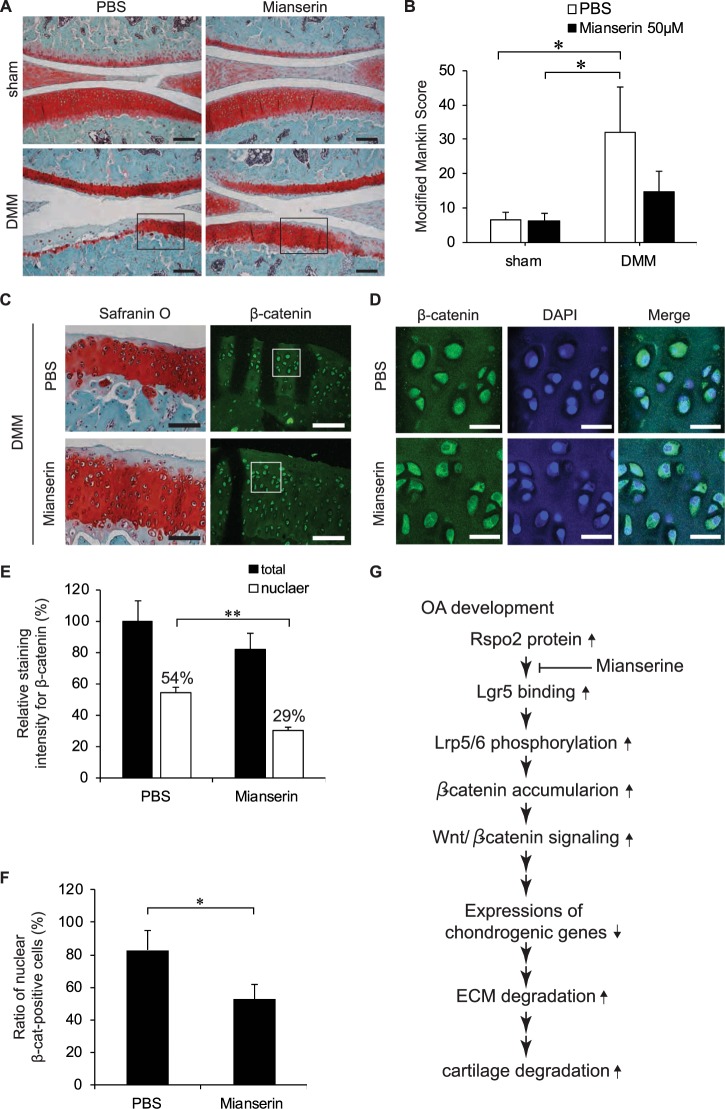
Mianserin ameliorates OA progression and reduces β-catenin expression in a rat model of OA. (**A**) Destabilization of medial meniscus (DMM) surgery was performed on the right knees of 6 Wistar/ST rats. Sham surgery was performed on the contralateral left knees by incising the skin and joint capsules. Fifty microliters PBS (*n* = 3) or 50 μM mianserin dissolved in 50 μl PBS (*n* = 3) was injected intraarticularly into both knees once weekly for 8 weeks after surgery. Representative histological sections of the medial compartments in the knee joints stained with Safranin-O and Fast-green are shown. (**B)** OA severity in the medial compartment of the knee joint was quantified by modified Mankin score at 8 weeks after the surgery. The sum of femoral and tibial scores is shown by mean and SD. *P < 0.05 by one-way ANOVA followed by Tukey’s *post-hoc* test. There was no statistical significance between PBS and mianserin in the DMM group (*P* = 0.079). (**C)** Left panels show magnifications of the boxed areas in **A**. Right panels show immunostaining with anti-β-catenin antibody in serial sections of the left panels. (**D)** Magnifications of the boxed areas in (**C**). Articular chondrocytes are stained with anti-β-catenin antibody and DAPI. (**E**,**F**) Blinded morphometry of β-catenin signals of the chondrocytes at the medial compartments with MetaMorph. (**E**) Signal intensities for β-catenin in the total cellular area and the DAPI-positive nuclear area are normalized to the number of cells and also to that in total cellular area in PBS-treated cartilage. **P < 0.01 by one-way ANOVA followed by Tukey’s *post-hoc* test. Mean and SD are indicated (*n* = 3 knees in each group). (**F**) The number of β-catenin-positive cells is divided by the number of DAPI signals to calculate the ratio of nuclear β-catenin-positive cells. The number of β-catenin-positive cells is divided by the number of DAPI signals to calculate the ratio of nuclear β-catenin-positive cells. *P < 0.05 by one-way ANOVA followed by Tukey’s *post-hoc* test. Scale bar = 200 μm (**A**), 100 µm (**C**), and 25 µm (**D**). (**G**) Model of Rspo2-mediated OA development, and the effect of mianserin.

We first confirmed that *Rnf43*/*Znrf3* mRNAs, *Lgr4-6* mRNAs, and Lgr5 protein were expressed in differentiated ATDC5 cells (Supplementary Fig. S2A,B). Rspo2 did not alter mRNA expressions of *Rnf43*/*Znrf3* (Fig. 3A,B) and *Lgr4/5/6* in 48 h in differentiated ATDC5 cells (Fig. 3C–E). In contrast, as in HEK293 cells^38^, Rspo2 increased the expressions of Lrp6, Lrp5, Frizzled6 (Fzd6), and β-catenin proteins in 48 h in differentiated ATDC5 cells (Fig. 3F and Supplementary Fig. S2C), which was likely to be initiated by increased phosphorylation at Ser1490 of Lrp6^39^ in 1.5 h (Fig. 3G). Mianserin suppressed rhRspo2-mediated increases of Lrp6, Lrp5, Fzd6, and β-catenin proteins, as well as Lrp6 phosphorylation, in differentiated ATDC5 cells and in HEK293 cells (Fig. 3F,G and Supplementary Fig. S2C,D). These observations prompted us to hypothesize that the target of mianserin is either upstream or on the cell membrane. Rspos activate Wnt/β-catenin signaling by forming a complex with the extracellular domains of both Lgr4/5/6 and RNF43/ZNRF3^17,18^. As Lgr5 was highly expressed in both OA cartilage (OAC) cells stated below and ATDC5 cells (Supplementary Fig. S2A,B), we evaluated the effect of mianserin on the binding of human Rspo2 to Lgr5 on the surface of HEK293 cells. We found that mianserin suppressed binding of Rspo2 to Lgr5 in a dose-dependent manner (Fig. 4H). We similarly evaluated the effect of mianserin on the binding of human Rspo2 to RNF43 on the surface of HEK293 cells, but observed no effect (Fig. 4H). Thus, mianserin directly suppresses binding of Rspo2 to Lgr5, and subsequently attenuates Lrp6 expression and β-catenin accumulation in chondrocytes.

### Mianserin inhibits β-catenin accumulation in chondrogenic cells in OA cartilage and mitigates articular cartilage degradation in a rat OA model

To examine the effects of mianserin on OA cartilage (OAC), we isolated OA cells from OA patients undergoing total knee replacement surgery. We first confirmed that *Rnf43*/*Znrf3* mRNAs, *Lgr4-6* mRNAs, and Lgr5 protein were expressed in OAC cells, as we observed in ATDC5 cells (Supplementary Fig. S2A,B). Cell viability assay showed that less than 100 µM of mianserin had no toxicity on OAC cells (Supplementary Fig. S3A). Immunoblotting showed that mianserin decreased rhRspo2-induced accumulation of β-catenin (Supplementary Fig. S3B,C), indicating that mianserin suppresses Wnt/β-catenin signaling in human OAC cells without overt toxicity.

We also examined the effects of mianserin on articular chondrocytes in a rat OA model. Rats were intraarticularly injected with 50 µl phosphate-buffered saline (PBS, control) or 50 µM mianserin once weekly for 8 weeks after OA induction by destabilization of the medial meniscus (DMM) surgery. At 8 weeks after surgery, mianserin improved Safranin-O staining on the articular surfaces and preserved articular cartilage structures in DMM-operated knees (Fig. 4A,B). We also observed that mianserin reduced the cellular and nuclear intensities of β-catenin (Fig. 4C–E), and the ratio of β-catenin-positive cells (Fig. 4F).

Thus, intraarticular injection of mianserin suppresses Wnt/β-catenin signaling in articular chondrocytes and ameliorates OA progression in a rat OA model (Fig. 4G). As the loss-of-function of β-catenin in cartilage also causes osteoarthritic changes by increasing apoptotic cells^40^, a certain level of Wnt/β-catenin signaling is required for articular cartilage^41^. Optimization of administration protocols, scrutinized analysis of adverse effects, and examination of an additive effect of concomitant treatment for a rat OA model need to be performed before mianserin is applied to human OA patients. Evaluation of the suppressive effect of long-term mianserin in depressed patients on OA development in epidemiological studies will also help elucidate the effect of mianserin on OA patients. In addition to OA, dysregulation of Rspo2 is also reported in several cancers^4,18,42^ and in proliferative diseases of bone and soft tissue^20,43^. We expect that mianserin is a potential repositioned drug that ameliorates human diseases associated with Rspo2-mediated abnormal activation of Wnt/β-catenin signaling.

## Materials and Methods

### Quantification of Rspo2 amounts in patient synovial fluid with knee OA

All studies involving human synovial fluid were performed in accordance with the protocol in Institutional Review Board of Nagoya University Hospital (The protocol No. 2015-0239-2) and approved by the Ethical Review Committee of the Nagoya University Graduate School of Medicine. After an appropriate written informed consent was obtained at Nagoya University Hospital, synovial fluid samples were acquired from 52 patients with knee OA (16 males and 36 females; ages, 36–93 years; and mean age, 74.6 years) with needle puncture at the outpatient clinic when the patients had hydrarthrosis. The diagnosis of knee OA was based on the criteria of the American Rheumatism Association^44^. Two authors independently evaluated plain knee radiographs and severity of knee OA was decided using the Kellgren-Lawrence (KL) radiographic grading system (grade 1, 14 patients; grade 2, 17 patients; grade 3, 13 patients; and grade 4, 8 patients)^29^. Each KL grade is characterized as follows: grade 1, doubtful OA with minimal osteophytes of dubious importance; grade 2, mild OA with definite osteophytes but unimpaired joint space; grade 3, moderate OA with moderate osteophytes and definite joint space narrowing; and grade 4, severe OA with considerably impaired joint space and sclerosis of subchondral bone^45^.

All patients had knee effusion caused by synovitis and underwent an arthrocentesis of the knee under aseptic condition. After centrifugation at 18,000 × *g* for 20 min at 4 °C to remove cells and other debris, concentrations of Rspo2 and total protein in the collected synovial fluid samples were quantified. Concentrations of Rspo2 were determined by the Human R-Spondin 2 (RSPO2) ELISA Kit (MyBioSource) according to the manufacturer’s instructions and using PowerScan4 (DS Pharma Biomedical) to measure absorbance at 450 nm. Concentrations of total protein were quantified by the Pierce 660 nm Protein Assay Kit (Pierce Biotechnology). We also evaluated concentrations of glycosaminoglycans (GAG) in synovial fluid by a spectrophotometric dye binding assay using 1,2, dimethylmethylene blue (DMMB; Santa Cruz Biotechnology)^46^. A positive correlation between GAG concentration in synovial fluid and KL grade was demonstrated in a previous report^28^.

### Screening of 1,271 FDA-approved drugs with TOPFlash luciferase reporter assay

We screened 1,271 FDA-approved chemical compounds (Prestwick Chemical) to identify a drug that specifically suppresses Rspo2-mediated activation of Wnt/β-catenin signaling. We quantified Wnt/β-catenin signaling activity with TOPFlash luciferase reporter assay. Human chondrosarcoma (HCS-2/8) cells were kindly provided by Dr. Masaharu Takigawa at Okayama University^47^. HCS-2/8 cells were cultured in Dulbecco’s Modified Eagle’s Medium (DMEM; Thermo Fisher Scientific) supplemented with 10% fetal bovine serum (FBS, Thermo Fisher Scientific). After 60–70% cellular confluency in a 100-mm dish, HCS-2/8 cells were transfected with 10 µg TOPFlash firefly luciferase reporter vector (M50 Super 8x TOPFlash plasmid, Addgene) and 0.25 µg Renilla luciferase plasmid (phRL-TK, Promega) using FuGENE 6 (Promega). At 24 h after transfection, the cells were seeded in a 96-well culture plate at 4 × 10^4^ cells/well and incubated for 24 h in the presence of 10 μM of each drug. Recombinant human Rspo2 protein (rhRspo2, R&D Systems) and/or recombinant human Wnt3a protein (rhWnt3a, R&D Systems) were added to the medium. Luciferase activity was measured with the Dual Luciferase Reporter Assay System (Promega) in PowerScan MX (DS Pharma Biomedical). Firefly luciferase activity was normalized by Renilla luciferase activity.

### Immunoblotting for β-catenin, total Lrp6, and phosphorylated Lrp6 in differentiated ATDC5 cells and human OAC cells

Differentiated ATDC5 cells and OAC cells were treated with 0–20 μM mianserin in the presence of 200 ng/ml rhRspo2 for the indicated times. Cells were harvested with ice-cold RIPA Lysis Buffer (Santa Cruz Biotechnology) with 0.1 mM dithiothreitol, 1 mg/ml leupeptin, 1 mM phenylmethylsulfonyl fluoride, 1 mg/ml aprotinin and phosphatase inhibitors (PhosSTOP; Sigma-Aldrich). Whole-cell lysates were separated on SDS-PAGE and transferred to a nitrocellulose membrane. Expression levels of β-catenin, β-actin, Lrp6, phosphorylated Lrp6, Lrp5, Frizzled 6 and Gapdh were determined using antibodies against β-catenin (dilution 1:1,000, 610154, Becton Dickinson), β-actin (1:250, sc47778, Santa Cruz Biotechnology), Lrp6 (1:500, sc15399, Santa Cruz Biotechnology), phospho-Lrp6 at Ser1490 (1:1,000, #2568, Cell Signaling Technology), Lrp5 (1:1,000, #5731, Cell Signaling Technology), Frizzled 6 (1:1,000, #5158, Cell Signaling Technology), and Gapdh (1:1,000, sc-47724, Santa Cruz Biotechnology), respectively. Signal intensities were normalized either to the β-actin or the total Lrp6 level. Three independent experiments were performed, and quantified using the NIH ImageJ software.

### Preparation of Rspo2-MycAP and cell surface binding of Rspo2 to Lgr5 and RNF43

The full-length human *RNF43* cDNA in pcDNA3-HA was kindly provided by Drs. Shigetsugu Hatakeyama and Tadasuke Tsukiyama at Hokkaido University, Japan^48^. We previously reported the full-length human *LGR5* (BC099650) cDNA in pcDNA3.1^49^. The C-terminal-deleted human *RSPO2* cDNA (amino acids 1–218) and the C-terminal of rat *Agrn* cDNA (amino acids 1141–1937 of M64780.1) were cloned into APtag-5 (GenHunter Corp.) at the HindIII and SnaBI sites to make the myc-and-alkaline phosphatase-tagged Rspo2 (Rspo2-mycAP) and the myc-and-alkaline phosphatase-tagged Agrn (Agrn-mycAP) as described previously^49^.

HEK293 cells were transfected with Rspo2-mycAP to make Rspo2-CM. Rspo2-mycAP and Agrn-mycAP in the CM were concentrated ~100-fold using Amicon Ultra-4 filters (Merck Millipore) for the cell surface binding assay. HEK293 cells were transfected with 3 μg RNF43/pcDNA3-HA, 5 μg Lgr5/pcDNA3.1, or 5 µg empty pcDNA3.1 using Lipofectamine 2000 (Thermo Fisher Scientific). Cells were incubated for 24 h with concentrated CM containing either Rspo2-mycAP or Agrn-mycAP then placed on ice for 1.5 h. Cells were washed with HABH buffer (0.5 mg/ml BSA, 0.1% NaN_3_, and 20 mM HEPES (pH 7.0) in Hank’s balanced salt solution), and then fixed in 60% acetone for 10 min on ice followed by 4% paraformaldehyde in 20 mM HEPES (pH 7.0) in Hank’s balanced salt solution for 10 min on ice. Fixed cells were washed once with 20 mM HEPES (pH 7.0) and 150 mM NaCl, incubated at 65 °C for 30 min, washed once with 0.1 M Tris-HCl (pH 8.0), and lysed with 0.1% Triton-X in Tris-HCl (pH 8.0). AP activity in the lysed solutions was measured using LabAssay ALP (Wako Pure Chemical Industries Ltd.) to quantify bound Rspo2-mycAP or Agrn-mycAP. Agrn-mycAP was used to confirm that it did not bind to the cell surface of RNF43-transfected or Lgr5-transfected HEK293 cells (data not shown).

### *In vivo* surgical induction of OA and intraarticular administration of mianserin

All animal experiments were performed in accordance with the recommendations in the Regulations on Animal Experiments in Nagoya University and approved by the Animal Care and Use Committee of the Nagoya University Graduate School of Medicine (The protocol No. 2017–29469). Ten-week-old male Wistar/ST rats were anesthetized with isoflurane and their right knee joints were induced to OA by surgical destabilization of the medial meniscus (DMM)^50^. Briefly, the knee joint was aseptically exposed with a medial capsular incision and the medial menisco-tibial ligament was resected to destabilize the medial meniscus. Sham surgery was performed on the contralateral left knee with incisions of the skin and joint capsule. Rats were randomly divided into PBS and mianserin groups (*n* = 3 each), and 50 µl PBS (control group) and 50 μM mianserin in 50 µl PBS (mianserin group) was intraarticularly injected into both knees once weekly, respectively. At 8 weeks after surgery, the rats were sacrificed and their knee joints were fixed overnight in 4% paraformaldehyde at 4 °C, decalcified, and embedded in paraffin. We made multiple sections around the middle portion of the medial femoral condyle (i.e. the weight-baring area) of each sample. A single section crossing the narrowest interarticular portion was stained with Safranin O and Fast-green. OA progressions were graded according to the modified Mankin histologic score^51^ on both tibial and femoral sides of articular cartilage.

The modified Mankin histological score is the sum of seven parameters. These include the articular cartilage structure (grades 0–11); tidemark duplication (grades 0–3); Safranin O staining (grades 0–8); fibrocartilage (grades 0–2); chondrocyte clones in uncalcified cartilage (grades 0–2); hypertrophic chondrocytes in calcified cartilage (grades 0–2); and subchondral bone (grades 0–2). Using a single well-cut and well-stained sagittal section crossing the narrowest interarticular portion in the medial compartment of the knee joint, the grades of OA were scored by a single blinded observer and averaged in each group of mice.

For immunofluorescence staining, the paraffin-embedded sections were deparaffinized and rehydrated. They were unmasked with 10 mM sodium citrate buffer (pH 6.0) at 90 °C for 15 min and incubated in 3% H_2_O_2_ for 15 min to inactivate endogenous peroxidases. They were then treated with a blocking buffer containing 5% goat serum in TBS-T for 1 h at room temperature and incubated with anti-β-catenin antibody (dilution 1:100; Cell Signaling Technology) at 4 °C overnight. The sections were then washed with TBS-T and incubated with Alexa 488-conjugated goat anti-rabbit IgG (dilution 1:500; Invitrogen) at room temperature for 1 h. The specimens were then mounted in VectaShield containing 2 µg/ml diamidino-2-phenylindole (DAPI) (Vector Laboratories, Inc.) and visualized with an A1Rsi microscope (Nikon Corp.). The average signal intensities of β-catenin in cytoplasm and nucleus of articular chondrocytes were blindly quantified with MetaMorph software (Molecular Devices, LLC). When the intensity of β-catenin in the nucleus was similar to or more than that in the cytoplasm, the cell was recognized as nuclear β-catenin-positive.

### Statistical analysis

All continuous variables are expressed as mean ± standard deviation (SD). Statistical significance was estimated either by Student’s *t*-test or one-way ANOVA followed by Tukey’s *post-hoc* test. *P*-values less than 0.05 was considered statistically significant. All statistical analyses were performed with SPSS ver. 23 (IBM Corp.).

### Other Materials and Methods

Details of other materials and methods are available in the Supplementary Information. This section includes detailed descriptions of total RNA extraction, real-time quantitative polymerase chain reaction (qPCR), Alcian blue staining, MTS assay, and isolation of human osteoarthritic chondrocyte (OAC) cells.

## Supplementary information

Supplementary information
