# Author Correction: Mianserin suppresses R-spondin 2-induced activation of Wnt/β-catenin signaling in chondrocytes and prevents cartilage degradation in a rat model of osteoarthritis

**DOI:** 10.1038/s41598-020-60112-4

**Published:** 2020-02-14

**Authors:** Toshiaki Okura, Bisei Ohkawara, Yasuhiko Takegami, Mikako Ito, Akio Masuda, Taisuke Seki, Naoki Ishiguro, Kinji Ohno

**Affiliations:** 1 Division of Neurogenetics, Center for Neurological Diseases and Cancer, Nagoya University Graduate School of Medicine, Nagoya, Japan; 2 Department of Orthopedic Surgery, Nagoya University Graduate School of Medicine, Nagoya, Japan

Correction to: *Scientific Reports* 10.1038/s41598-019-39393-x, published online 26 February 2019

This Article contains errors in the Reference list. Reference 21 is incorrectly listed as:

Masahiro N. *et al*. A genome-wide association study identifies susceptibility loci for ossification of the posterior longitudinal ligament of the spine. *Nature Genetics* **46**(9), 1012–1016 (2014).

The correct reference is listed below as ref. 1.

Additionally, Reference 23 is incorrectly listed as:

Rita D. Hypertrophic differentiation of chondrocytes in osteoarthritis: the developmental aspect of degenerative joint disorders. *Arthritis Research & Therapy* **12**(5), 216 (2010).

The correct reference is listed below as ref. 2.

Furthermore, Reference 25 is incorrectly listed as:

Marlies, B. *et al*. The Reason for Discontinuation of the First Tumor Necrosis Factor (TNF) Blocking Agent Does Not Influence the Effect of a Second TNF Blocking Agent in Patients with Rheumatoid Arthritis. *The Journal of Rheumatology* **36**(10), 2171–2177 (2009).

The correct reference is listed below as ref. 3.

In addition, there is an error in the legend of Figure 4, where:

“The number of β-catenin-positive cells is divided by the number of DAPI signals to calculate the ratio of nuclear β-catenin-positive cells. The number of β-catenin-positive cells is divided by the number of DAPI signals to calculate the ratio of nuclear β-catenin-positive cells. *P < 0.05 by one-way ANOVA followed by Tukey’s *post-hoc* test. Scale = bar 200 μm (**A**), 100 µm (**C**), and 25 µm (**D**). (**G**) Model of Rspo2-mediated OA development, and the effect of mianserin.”

should read:

“The number of β-catenin-positive cells is divided by the number of DAPI signals to calculate the ratio of nuclear β-catenin-positive cells. *P < 0.05 by one-way ANOVA followed by Tukey’s *post-hoc* test. Scale bar = 200 μm (**A**), 100 µm (**C**), and 25 µm (**D**). (**G**) Model of Rspo2-mediated OA development, and the effect of mianserin.”
