# Supplementary material for: Mianserin suppresses R-spondin 2-induced activation of Wnt/β-catenin signaling in chondrocytes and prevents cartilage degradation in a rat model of osteoarthritis
Source: Sci Rep. 2019 Feb 26;9:2808. doi: 10.1038/s41598-019-39393-x (PMC6391487; doi:10.1038/s41598-019-39393-x)

Mianserin suppresses R-spondin 2-induced activation of Wnt/β-catenin signaling in chondrocytes and prevents cartilage degradation in a rat model of osteoarthritis

Toshiaki Okura[1, 2], Bisei Ohkawara[1]*, Yasuhiko Takegami[2], Mikako Ito[1], Akio Masuda[1], Taisuke Seki[2], Naoki Ishiguro[2], Kinji Ohno[1]

[1]Division of Neurogenetics, Center for Neurological Diseases and Cancer, Nagoya University Graduate School of Medicine, Nagoya, Japan
[2]Department of Orthopedic Surgery, Nagoya University Graduate School of Medicine, Nagoya, Japan

*Address correspondence to Bisei Ohkawara, Division of Neurogenetics, Center for Neurological Diseases and Cancer, Nagoya University Graduate School of Medicine, 65 Tsurumai, Showa-ku, Nagoya 466-8550, Japan.
Email: biseiohkawara@med.nagoya-u.ac.jp

Keywords
Mianserin; R-spondin 2 (Rspo2); Wnt/β-catenin signaling; Osteoarthritis

Supplementary Information

Supplementary materials and methods

Total RNA extraction and real-time quantitative PCR analysis

Total cellular RNA was extracted using Trizol, and the first strand cDNA was synthesized with oligo-dT primer (Thermo Fisher Scientific) and ReverTra Ace (Toyobo). Quantitative real-time PCR (qRT-PCR) was performed using LightCycler 480 Real-Time PCR (Roche) and SYBR Green (Takara). The expression level of a specific gene was normalized for that of *Gapdh*. Primer sequences are shown in Supplementary Table S2.

Alcian blue staining

ATDC5 mouse chondrogenic progenitor cells (Riken BioResource Center, Japan) were cultured in DMEM/F12 (a mixture of DMEM and Ham's F12 medium, Sigma-Aldrich) supplemented with 5% fetal bovine serum (FBS, Thermo Fisher Scientific). To induce chondrogenic differentiation, the cells were added with 1% insulin-transferrin-sodium selenite (ITS, Gibco) for 2 weeks. Differentiated ATDC5 cells were treated with 0, 2.5, 5, 10 and 20 μM mianserin in the presence of 200 ng/ml rhRspo2 or 90 ng/ml rhWnt3a for 48 h. Cells were fixed with methanol for 30 min at −20°C and stained overnight with 0.5% Alcian Blue 8 GX (Sigma-Aldrich) in 1 N HCl. For quantitative analyses, Alcian blue-stained cells were lysed in 200 μl of 6 M guanidine HCl for 6 h at room temperature, and the optical density of the extracted dye was quantified at 620 nm by PowerScan4 (DS Parma Biomedical).

MTS assay

Cell proliferation of human OAC cells was quantified by the MTS assay (Cell 96 AQueus One Solution Cell Proliferation Assay, Promega) according to the manufacturer's instructions. Briefly, OAC cells were seeded at 6×10^4/well in a 96-well culture plate with CellTiter 96 AQueous One Solution Reagent and incubated for 2 h in the presence of variable concentrations of mianserin. Then, absorbance was measured at 490 nm using an absorbance microplate reader (Sunrise Remote, Tecan).

Isolation of human osteoarthritic chondrocyte (OAC) cells

All studies using human OAC cells were performed in accordance with the recommendations in Institutional Review Board of Nagoya University Hospital and approved by the Ethical Review Committee of the Nagoya University Graduate School of Medicine. After written informed consent was obtained at Nagoya University Hospital, human OAC cells were isolated from the dissected cartilage of patients who had undergone joint replacement surgery for knee OA as the method described previously [10]. This protocols involved in the use of human tissues is reviewed and approved by Institutional Review Board (The protocol no. 2015-0239-2). OAC cells were cultured in DMEM with 10% FBS, penicillin (100 U/ml), and streptomycin (100 mg/ml) at 37 °C in a 5% CO_2 atmosphere. At confluence, the cells were detached and seeded in a 96-well plate for MTS assay or a 12-well plate for Western blotting.

Supplementary Table S1. Rspo2 and GAG levels for individual patients.

Pt. no	Age (year)	Sex	KL score	Rspo2 concentration (pg/ml)	Rspo2 in total protein (pg/mg)	GAG concentration (mg/ml)
1	79	female	1	6.6	0.5	91
2	93	female	1	5.5	0.6	102
3	62	female	1	14.1	1.4	220
4	74	female	1	21.3	1.3	272
5	74	female	1	20.0	1.1	348
6	85	female	1	19.4	0.9	213
7	67	female	1	17.5	0.8	340
8	68	female	1	21.5	1.1	36
9	48	female	1	17.5	1.0	249
10	77	female	2	13.0	0.5	14
11	82	female	2	20.1	0.8	15
12	72	female	2	26.0	2.2	1133
13	91	female	2	59.3	3.0	392
14	74	female	2	36.3	1.0	179
15	84	female	2	32.0	0.8	2680
16	77	female	2	18.4	1.3	1155
17	75	female	2	51.7	1.7	304
18	69	female	2	18.4	0.7	282
19	56	female	2	11.3	0.5	443
20	83	female	2	0.4	0.1	421
21	75	female	2	16.6	0.8	62
22	77	female	2	14.1	4.5	91
23	82	female	3	33.8	2.1	1221
24	71	female	3	7.3	0.9	788
25	75	female	3	3.0	0.7	355
26	77	female	3	31.2	3.8	0
27	75	female	3	21.3	1.6	395
28	83	female	3	63.7	4.8	290

29	82	female	3	66.2	4.8	285
30	83	female	3	87.5	4.0	191
31	91	female	4	17.5	0.4	1122
32	74	female	4	9.0	0.3	821
33	72	female	4	5.5	0.6	1457
34	81	female	4	2.1	0.5	1396
35	91	female	4	2.1	0.1	1158
36	80	female	4	104.6	2.6	1249
37	86	male	1	15.8	0.5	397
38	75	male	1	14.1	0.4	168
39	91	male	1	19.2	0.4	148
40	36	male	1	2.9	0.7	201
41	56	male	1	4.7	0.2	293
42	84	male	2	14.7	1.3	55
43	67	male	2	9.8	0.8	553
44	81	male	2	6.4	0.5	872
45	68	male	2	4.7	0.6	597
46	55	male	3	26.0	1.3	476
47	91	male	3	13.8	1.0	1617
48	54	male	3	13.2	0.7	572
49	84	male	3	11.3	1.3	146
50	81	male	3	< 0.1	< 0.1	2075
51	55	male	4	32.9	1.3	1289
52	55	male	4	18.4	0.5	1223

Pt. no., Patient serial number; KL., Kellgren-Lawrence; GAG., glycosaminoglycans

Supplementary Table S2. Primer sequences for qRT-PCR

Gene	Primer sequence
Human *AXIN2*	For. 5’ ctccccaccttgaatgaaga 3’
	Rev. 5’ gtttccgtggacctcacact 3’
Human *CCND1*	For. 5’ ccgtccatgcggaagatc 3’
	Rev. 5’ gaagacctcctcctcgcact 3’
Human *MYC*	For. 5’ aatgaaaaggcccccaaggtagttatcc 3’
	Rev. 5’ gtcgtttccgcaacaagtcctcttc 3’
Human *RNF43*	For. 5’ agcatgagtggtggccaccag 3’
	Rev. 5’ atctcacacagcctgttcac 3’
Human *ZNRF3*	For. 5’ catcgtcaacaagcagaaagtg 3’
	Rev. 5’ ggagaccacgacgaagaaag 3’
Human *LGR4*	For. 5’ ctttgtttgccatttccta 3’
	Rev. 5’ ctagtgagtttaatagcactaa 3’
Human *LGR5*	For. 5’ tcagtcagctgctcccgaat 3’
	Rev. 5’ cgtttcccgcaagacgtaac 3’
Human *LGR6*	For. 5’ cacaccccagtgtccagtgtag 3’
	Rev. 5’ ccacaggaaatgccagtcaag 3’
Mouse *Axin2*	For. 5’ gcagcagatccgggaggatgaa 3’
	Rev. 5’ gattgacagccgggggtcttga 3’
Mouse *Sox9*	For. 5’ tatgtggatgtgtgcgtgtg 3’
	Rev. 5’ ccagccacagcagtgagtaa 3’
Mouse *Col2a1*	For. 5’ ggaatttggtgtggacataggg 3’
	Rev. 5’ ggtcaggtcagccattcagt 3’
Mouse *Acan*	For. 5’ cctcgggcagaagaaaga 3’
	Rev. 5’ gtctcatgctccgcttctgt 3’
Mouse *Rnf43*	For. 5’ aggcagaggaggcacaactac 3’
	Rev. 5’ gccaactttctgctccactc 3’
Mouse *Znrf3*	For. 5’ ctgaacttctgggcttctgg 3’
	Rev. 5’ gccaactttctgctccactc 3’
Mouse *Lgr4*	For. 5’ agtcctaaccctccagaacaatcag 3’

	Rev. 5’ggtaatatggttggcatctaagcg 3’
Mouse *Lgr5*	For. 5’ agccttcaatccctgcgcctag 3’
	Rev. 5’ gacagggacgtctgtgagagcattg 3’
Mouse *Lgr6*	For. 5’ accacctgcgcttcctggaggag 3’
	Rev. 5’ cagctcccatagtgcctctgctg 3

For., forward primer; Rev., reverse primer

Figure Legends

Supplementary Figure S1. RhRspo2 enhances the TOPFlash reporter activity in the presence of a low dose of rhWnt3a. (A) HCS-2/8 human chondrosarcoma cells transfected with TOPFlash were treated with indicated concentrations of recombinant human Rspo2 (rhRspo2) and recombinant human Wnt3a (rhWnt3a) for 24 h. Firefly luciferase activity was normalized to the TK promoter-driven Renilla luciferase activity, and expressed as relative luciferase activities. Mean and SD are indicated (n = 3). **(B-D)** HCS-2/8 human chondrosarcoma cells were treated with 120 ng/ml rhRspo2 and 20 ng/ml rhWnt3a for 24 h with or without 10 μM mianserin. Expression levels of each mRNA quantified by qRT-PCR were normalized for those of *Gapdh* and also for untreated cells. Means and SD are indicated (n = 3). *$P < 0.05$ and **$P < 0.01$ by one-way ANOVA followed by Tukey's *post-hoc* test.

Supplementary Figure S2. Mianserin reduces Rspo2-induced phosphorylation of Lrp6 in HEK293 cell. (A) Human osteoarthritic chondrocyte (OAC) cells or ATDC5 cells were cultured and harvested. Expression levels of each mRNA quantified by qRT-PCR were normalized for those of *Gapdh*. Means and SD are indicated (n = 3). **(B)** Human OAC cells or ATDC5 cells were cultured and harvested. Representative Western blots with antibodies against Lgr5 and Gapdh are shown. **(C)** Differentiated ATDC5 cells were cultured with 10 μM mianserin and 200 ng/ml rhRspo2 for 48 h. Representative Western blots and intensities of Frizzled6 (Fzd6), Lrp5, and Lrp6 normalized for those of β-actin and also for untreated cells are shown. Mean and SD are indicated (n = 3). **(D)** HEK293 cells were treated with 10 μM mianserin and 200 ng/ml rhRspo2 for 1.5 h. Representative Western blots are shown. Densitometric ratios of

phospho-Lrp6/Lrp6 were normalized for that of untreated control cells. Mean and SD are shown (n = 3). *$P < 0.05$ by one-way ANOVA followed by Tukey's *post-hoc* test.

Supplementary Figure S3. Mianserin decreases β-catenin levels in human osteoarthritic chondrocyte (OAC) cells. (A) OAC cells were treated with CellTiter 96 AQueous One Solution Reagent for 2 h in the presence of the indicated mianserin concentrations. The number of cells was estimated from the absorbance at 490 nm (n = 6). Absorbance was normalized for that without mianserin. **(B, C)** Human OAC cells were treated with conditioned medium containing 200 ng/ml rhRspo2 for 48 h in the presence of the indicated concentrations of mianserin. Representative Western blots **(B)** and relative intensities of β-catenin normalized for those of β-actin and also for untreated cells **(C)** are shown. Mean and SD are indicated (n = 3) **$P < 0.01$ by one-way ANOVA followed by Tukey's *post-hoc* test.

Supplementary Figure S4. Full length images of the cropped gels. Full length images of the cropped gels presented in main Figure 3F **(A)**, Supplementary Figure 2B **(B)**, Supplementary Figure 2C **(C)**, in main Figure 3G **(D, right panel)**, Supplementary Figure 2D **(D, left panel)**, and Supplementary Figure 3**(E)**.

Supplementary Figure S1

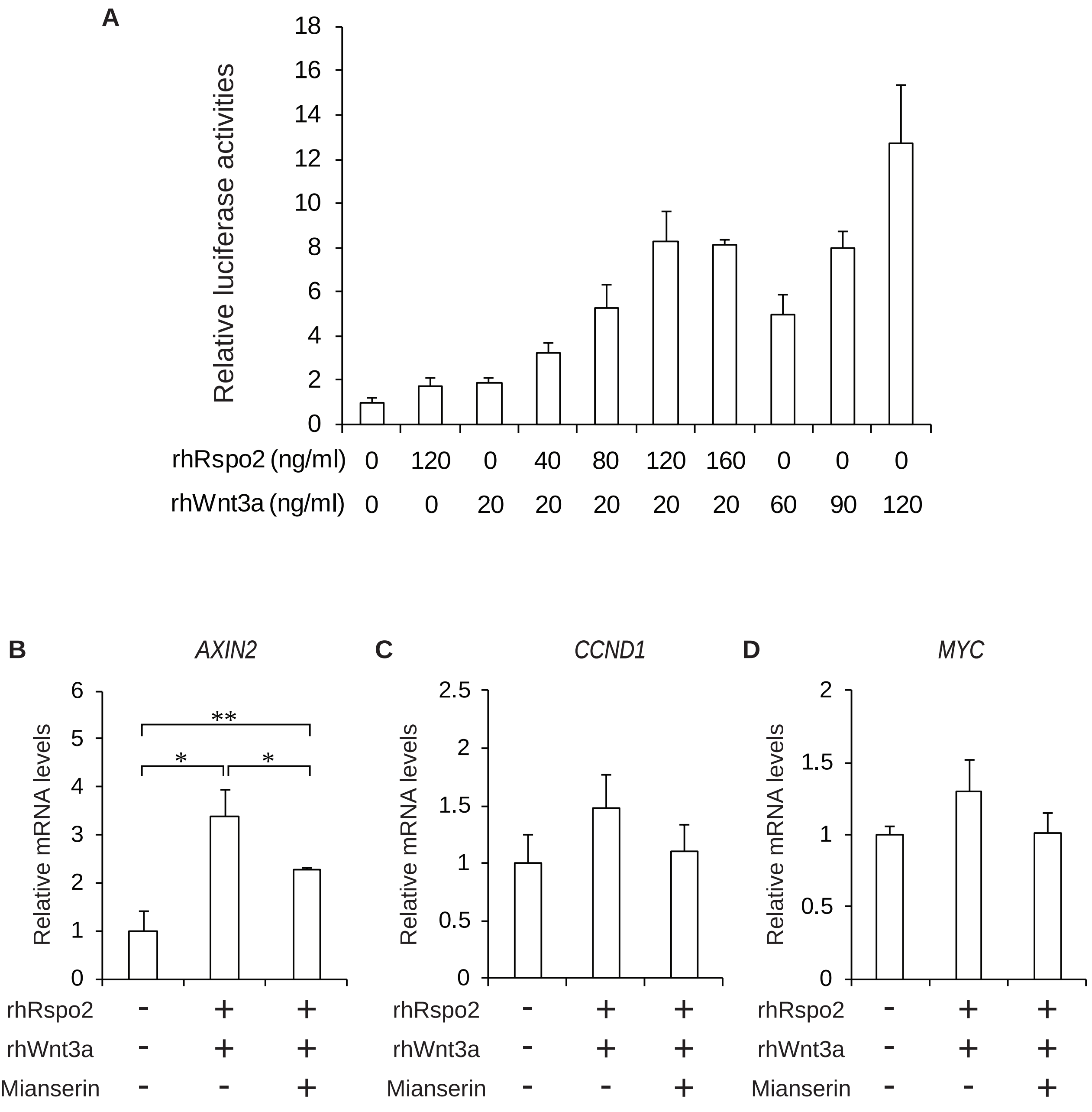

Supplementary Figure S2

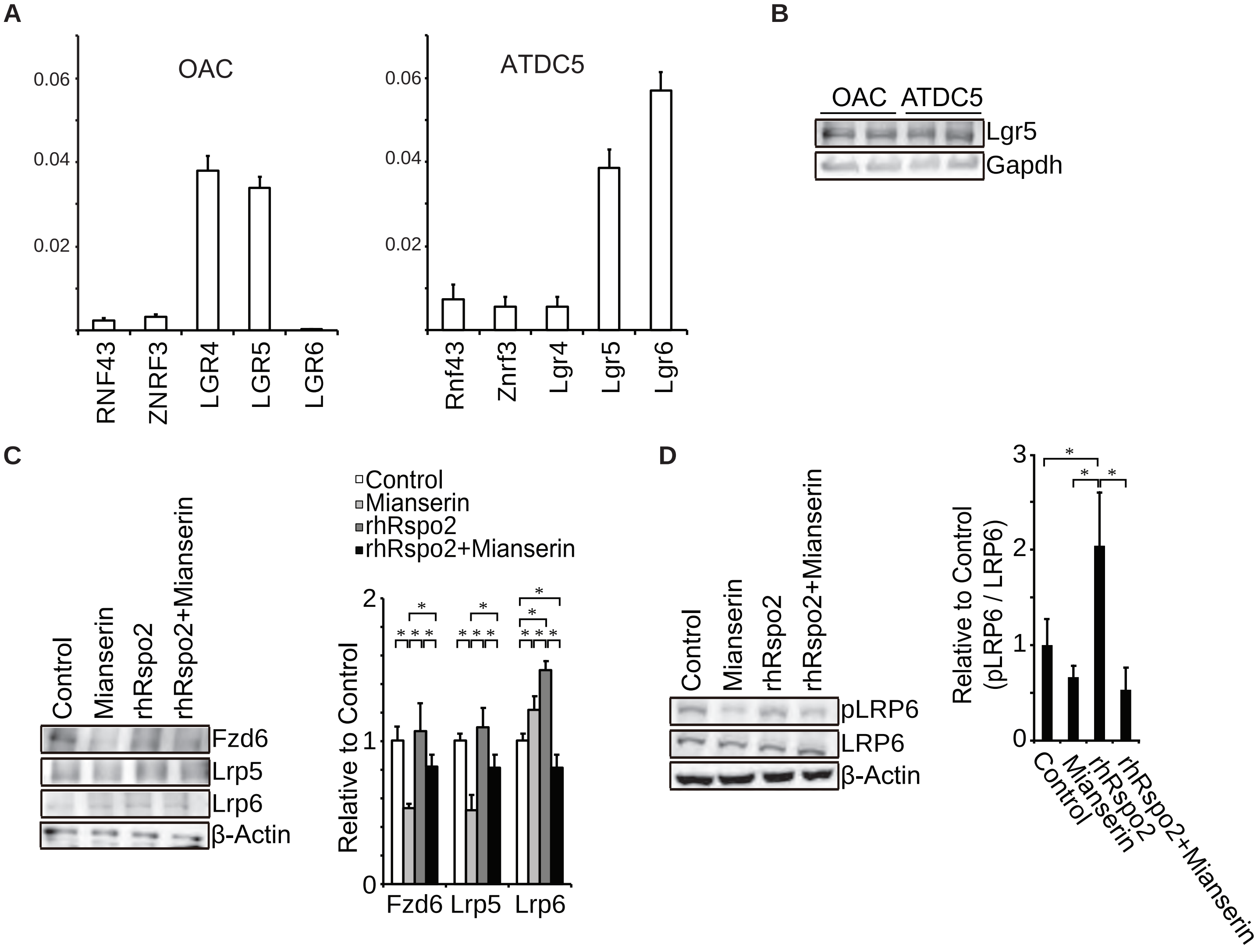

Supplemetary Figure S3

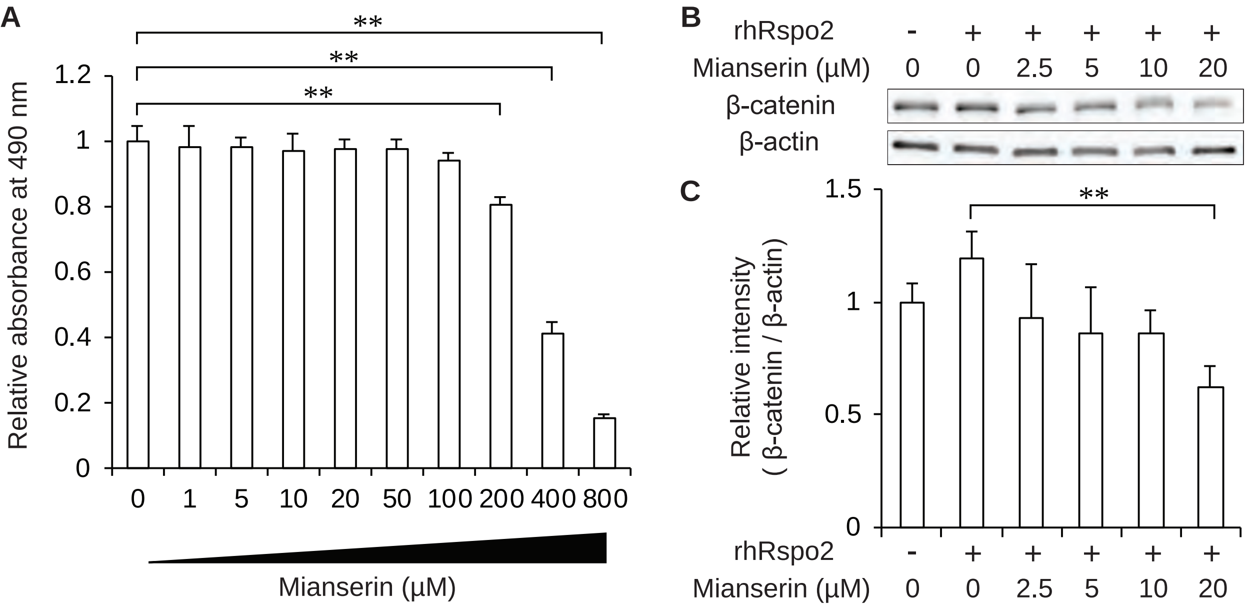

Supplementary Figure S4

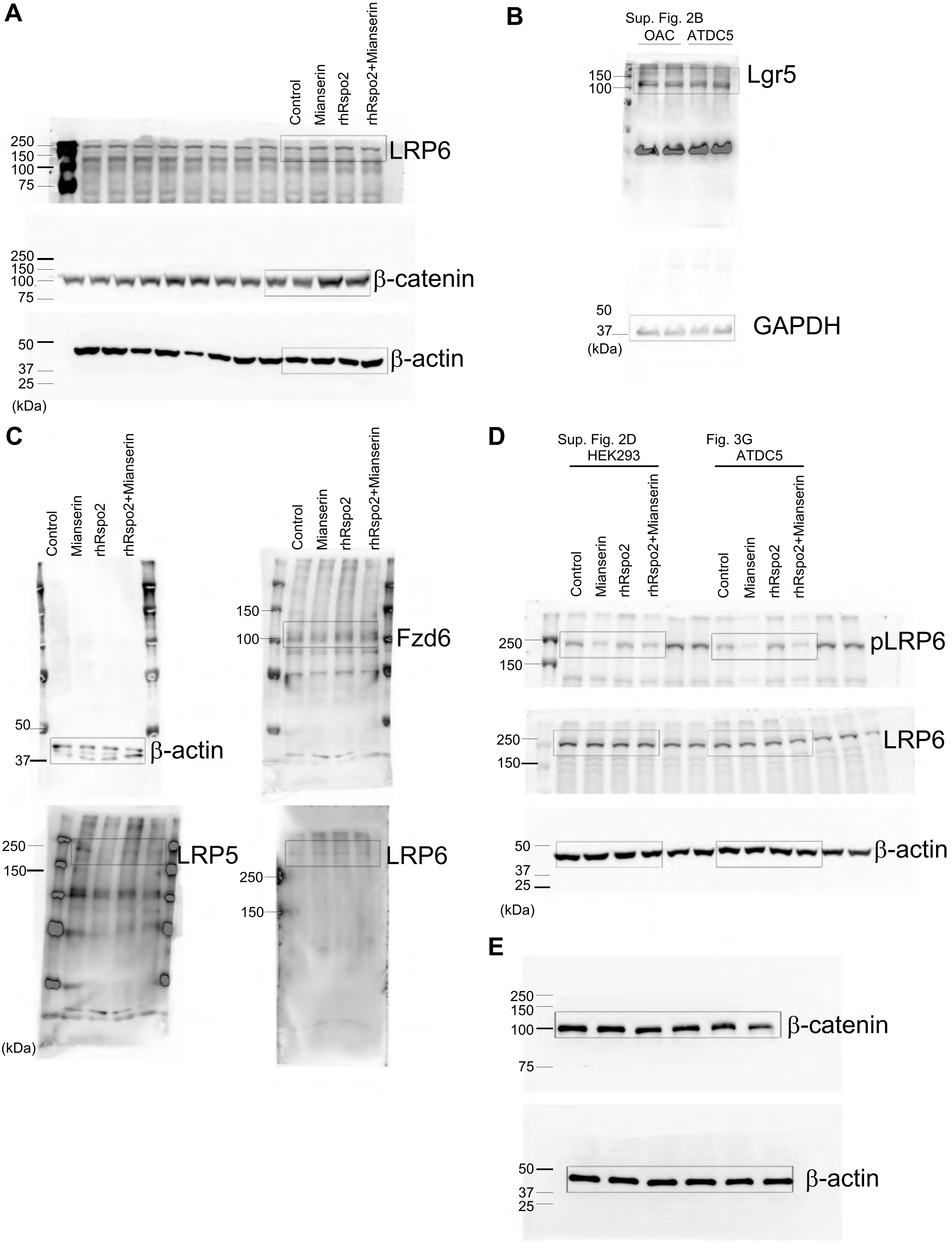

Supplement: Supplementary file 1 — Supplementary information [file 41598_2019_39393_MOESM1_ESM.pdf]
